# *S*-Nitrosation and Ubiquitin-Proteasome System Interplay in Neuromuscular Disorders

**DOI:** 10.1155/2014/428764

**Published:** 2014-01-30

**Authors:** Salvatore Rizza, Costanza Montagna, Giuseppina Di Giacomo, Claudia Cirotti, Giuseppe Filomeni

**Affiliations:** ^1^Department of Biology, University of Rome “Tor Vergata”, 00133 Rome, Italy; ^2^Research Center, IRCCS San Raffaele Pisana, 00166 Rome, Italy

## Abstract

Protein *S*-nitrosation is deemed as a prototype of posttranslational modifications governing cell signaling. It takes place on specific cysteine residues that covalently incorporate a nitric oxide (NO) moiety to form *S*-nitrosothiol derivatives and depends on the ratio between NO produced by NO synthases and nitrosothiol removal catalyzed by denitrosating enzymes. A large number of cysteine-containing proteins are found to undergo *S*-nitrosation and, among them, the enzymes catalyzing ubiquitination, mainly the class of ubiquitin E3 ligases and the 20S component of the proteasome, have been reported to be redox modulated in their activity. In this review we will outline the processes regulating *S*-nitrosation and try to debate whether and how it affects protein ubiquitination and degradation via the proteasome. In particular, since muscle and neuronal health largely depends on the balance between protein synthesis and breakdown, here we will discuss the impact of *S*-nitrosation in the efficiency of protein quality control system, providing lines of evidence and speculating about its involvement in the onset and maintenance of neuromuscular dysfunctions.

## 1. Redox Modifications and Cell Signaling

The main molecular mechanism underlying signal transduction in eukaryotic cells relies on the regulation of protein function by posttranslational modifications. The transient and reversible attachment of reactive moieties on specific residues is able to induce a plethora of effects on protein function, thereby giving rise to a dynamic interplay of protein interactions capable to convey signals within the cell. Among these, redox signal is highly specific and represents a prerogative of sulfur-containing residues. In particular, cysteine is more versatile than methionine in forming adducts because of its capability to be present under numerous oxidation states [1]. This feature allows cysteine reacting with many oxidant species, such as hydrogen peroxide, glutathione disulfide (GSSG), or nitric oxide (NO) moiety, generating in such a way the reversible *S*-hydroxylated (SOH), *S*-glutathionylated (*S*SG), or *S*-nitrosated (*S*NO) derivative, respectively [2]. Among them, glutathionylation or, widely, disulfide bond formation is the most stable cysteine oxidative modification. This is the reason why, physiologically, both *S*-hydroxylated and *S*-nitrosated proteins usually convert in *S*-glutathionylated (in the presence of high concentrations of GSH, as normally occurs inside the cells) or, widely, disulfide adducts. Therefore, hydrogen peroxide and NO can indirectly induce cysteine oxidation to disulfide. It should be also reminded that, similarly to hydrogen peroxide, NO overproduction has been reported being associated with irreversible sulfhydryl oxidation, such as sulfinylation (*S*O_2_H) or sulfonylation (*S*O_3_H) of metalloproteases [3]. However, these last modifications likely imply the production of peroxinitrite (ONOO^−^), a more dangerous and more oxidant reactive nitrogen species (RNS) generated by the reaction between NO and superoxide anion (O_2_^∙−^), which has been copiously reported being involved in protein tyrosine nitration (see below).

### 1.1. Implication of NO in Cell Signaling

Nitric oxide is a gaseous and membrane-diffusible radical molecule produced by the class of NADPH-dependent enzymes NO synthase (NOS) [4, 5]. The first evidence of NO involvement in signal transduction goes back to 1983 when it was demonstrated that cGMP-mediated regulation of blood vessel tone depended on the direct binding of NO to the heme iron (Fe-nitrosylation) of guanylyl cyclase [6, 7]. Since then, increasingly data provided further and indisputable lines of evidence pointing out this pathway being, in fact, implicated in many other functions, such as immune response [8], neurotransmission [9], and mitochondrial respiration [10]. Indeed, physiologically NO can bind to free iron within any heme-containing protein with a free ligand position [11]. By this reaction, and also by binding the copper binuclear centers in a noncompetitive manner [12], NO can regulate cytochrome *c* oxidase activity and, more widely, it can tune mitochondrial respiration.

**Figure 1:**
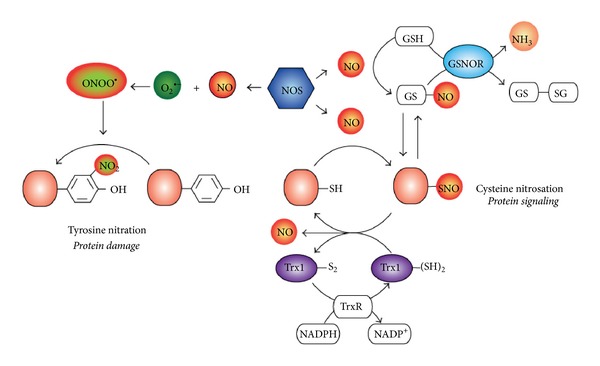
Tyrosine nitration versus *S*-nitrosation. Nitric oxide (NO), produced by NO synthase (NOS), can affect protein structure and function in different ways. Here only posttranslational modifications directly modifying protein residues are shown, tyrosine nitration (right) and cysteine *S*-nitrosation (left). The former adduct is irreversible (so far, no denitrating enzyme has ever been found) and responsible for protein damage occurring mostly upon the overproduction of NO. Indeed, under this condition (called nitrosative stress) NO can rapidly react with superoxide anion (O_2_^∙−^) to form peroxinitrite (ONOO^−^) which is the main harmful radical species inducing tyrosine nitration. Conversely, upon physiological production of NO, reactive cysteines of both redox-sensitive proteins and glutathione (GSH) can undergo *S*-nitrosation, thereby generating their *S*-nitrosothiol derivatives, Prot-SNOs and *S*-nitrosoglutathione (GSNO), respectively. Prot-SNOs and GSNO are in equilibrium by transnitrosation reactions; therefore, the GSNO catabolizing enzyme, GSNOR reductase (GSNOR), by regulating GSNO levels also impacts on protein nitrosation extent. Thioredoxin 1 (Trx1) also participates in protein denitrosation by means of its vicinal thiols that reduce Prot-SNO and oxidize to an internal disulfide bridge, whose further reduction is catalyzed by Trx reductase (TrxR) and ensured by reducing equivalents provided by NADPH. Although both GSNOR and Trx1 concur to modulated protein *S*-nitrosation, it should be reminded that the former enzyme completely reduces GSNO to glutathione disulfide (GSSG) and ammonia (NH_3_), whereas the latter releases the NO moiety of Prot-SNOs as NO itself or nitroxyl anion (HNO), which are species still capable to target protein substrates.

Alongside these findings, the involvement of NO in redox signaling was emerging and progressively assuming distinctive signatures. Over those years, it became clear, indeed, that *S-*nitrosation is the main reversible posttranslational modification induced by NO able to regulate different classes of proteins [13–15]. The discovery of denitrosating enzymatic systems, namely, those dependent on thioredoxin 1 (Trx1) and *S*-nitrosoglutathione reductase (GSNOR) activities, which actively participate the *S*-nitrosothiol-to-sulfhydryl (SNO-to-SH) reduction [16–19] (Figure 1), definitively sealed the importance of *S*-nitrosation in cell physiology and human health (Table 1).

Actually, NO can also trigger irreversible modifications to proteins, such as the formation of nitrotyrosine. However, this is an event occurring only under certain conditions (nitrosative stress) that do not deal with signaling but just represents marker of damage. In particular, when NO is overproduced and no longer neutralized, it can react with oxygen-derived radical and nonradical species (ROS) thereby generating more dangerous RNS, for example, ONOO^−^ [20, 21] (Figure 1).

As previously mentioned, protein *S*-nitrosothiols (PSNOs) generation is specific, redox-mediated, and reversible [22] depending on several factors, such as the environmental hydrophobicity conditions and the steric hindrance, as well as the net charge and the presence of oxygen [2, 13]. In addition, it has been reported that the specificity of *S-*nitrosation can also depend on the presence of an *S-*nitrosation motif [23] roughly characterized by acid-base residues surrounding the target cysteine [24]. Nevertheless, many other factors deeply impact on the identity of specific proteins undergoing *S*-nitrosated, such as cellular localization and compartmentation of NO production [25]. In addition, the evidence that NO moiety can be also transferred among proteins or low-molecular-weight *S*-nitrosothiols (e.g., *S*-nitrosoglutathione, GSNO), adds further complexity to the regulation of protein *S*-nitrosation [26].

### 1.2. Transnitrosation and Denitrosation

The major determinant for NO transfer (transnitrosation) is the difference between the redox potential of the two interacting cysteine residues [27]. This aspect takes more importance if transnitrosation does not occur with low-molecular-weight thiols, but takes place between proteins. Indeed, this reaction is responsible for the propagation of many NO-mediated cell signaling pathways and its significance has been highlighted quite recently in many physiopathological processes, such as (i) NO exchange between hemoglobin and the anion exchanger 1, which mediates NO release from erythrocytes [28]; (ii) transnitrosation among thioredoxin (Trx), caspase-3 and the inhibitor of apoptosis (IAPs) proteins, which is involved in the regulation of cell death by apoptosis [27, 29–31]; (iii) glyceraldehyde 3-phosphate dehydrogenase (GAPDH)-mediated *S*-nitrosation of nuclear proteins, which contributes to cell death and accounts for the pathogenesis of several neurodegenerative diseases [32, 33]; (iv) transnitrosation between the neuronal-specific cyclin dependent kinase 5 (Cdk5) and dynamin-related protein 1 (Drp1), which plays a pivotal role in mitochondrial dysfunction typical of neurodegenerations [26, 34].

PSNOs levels are counterbalanced by denitrosation systems, the most important of which are the glutathione (GSH)/GSNOR and the Trx1/Trx reductase (TrxR) couples [2, 18, 35, 36] (Figure 1). In the light of what mentioned above, only a specific subset of proteins is *S*-nitrosated, resulting in the selective modulation of specific signaling pathways. In this scenario, it is plausible that the propagation or modulation of cell signals by *S*-nitrosation often implies a crosstalk with signaling modalities mediated by other mechanisms of posttranslational modification [37]. In the last decades, the discovery of *S*-nitrosation of both protein kinases and phosphatases suggested the influence of this modification in a wide range of signal transduction pathways mediated by phosphorylation/dephosphorylation [38–40]. This aspect is of great importance if one considers that *S*-nitrosation can convert into *S*-glutathionylated/disulfide adduct and that this is a well-known mechanism driving signal transduction mediated by phosphorylative cascades [41]. Likewise, it has emerged that *S*-nitrosation may also operate in the nucleus on epigenetic mechanisms of transcriptional regulation, in particular by interfering with histone acetylation status [42]. Ubiquitination of proteins, which represents the pivotal reaction underlying protein turnover and quality control, might be also affected by *S*-nitrosation because many enzymes involved in ubiquitination process have critical cysteines, which have been reported undergoing oxidation [43–45] or, actually, sumoylation [46]. *S*-nitrosation could therefore have deep implications in a number of pathophysiological conditions involving ubiquitin-proteasome system (UPS).

## 2. Dual Role of *S*-Nitrosation in Ubiquitin-Proteasome System

### 2.1. Ubiquitination

Ubiquitination is an enzymatic posttranslational modification process occurring on proteins, based on the ligation of ubiquitin (an 8.5 kDa protein) on a target protein lysine residue (monoubiquitination). This first step may be followed by the formation of ubiquitin chains through the attachment of additional ubiquitin moieties to one or more of the seven lysine residues within conjugated ubiquitin (polyubiquitination). The covalent attachment of ubiquitin on lysine residues requires the coordinated reaction of three enzymes. The first reaction is accomplished by the ubiquitin-activating enzymes (E1), which promote ubiquitin adenylation required for its covalent binding to the cysteine residue located at the E1 active site. Ubiquitin-charged E1 enzymes next transfer ubiquitin to the cysteine of the ubiquitin-conjugating enzymes (E2) that, in concert with a wide class of enzymes known as ubiquitin ligases (E3) needed for target recognition, finally transfer ubiquitin on lysine residues of specific substrates [47]. Protein ubiquitination is the major mechanism underlying protein turnover and quality control, as it is responsible for the redirection of damaged/unfolded proteins towards the proteasome to be degraded. In addition, it mediates the recognition of damaged organelles, or protein aggregates, by means of adaptor proteins (i.e., p62/sequestosome) that are also implicated in autophagy-mediated degradation [48]. This further function emphasizes the paramount role of ubiquitination in cellular homeostasis, as it is at the crossroads between different degradative pathways (both proteasome and autophagy mediated). Although ubiquitination was initially shown to drive protein degradation, it is now commonly accepted that the nature of ubiquitination (monoubiquitination versus polyubiquitination), as well as the type of interubiquitin linkages in polyubiquitin chains, mediates different response in cellular processes and signaling, including antigen processing [49], apoptosis [50], cell cycle [51], DNA transcription, and repair [52].

### 2.2. Inhibitory Effects of *S*-Nitrosation

**Figure 2:**
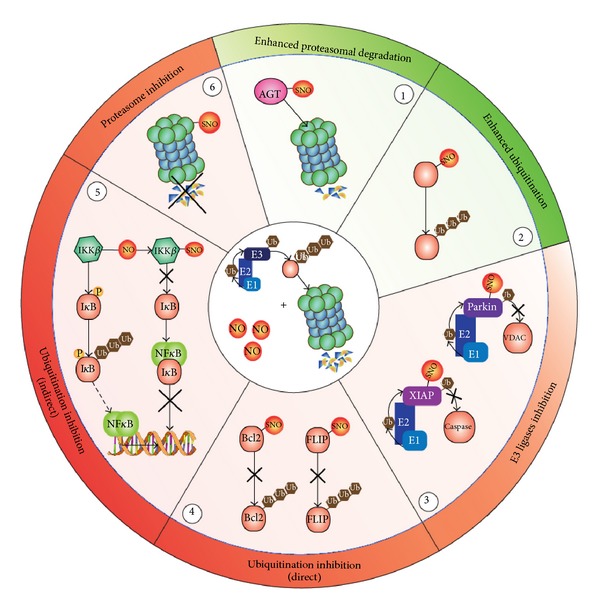
*S*-nitrosation-induced cellular effects on ubiquitin-proteasome system. Nitric oxide (NO) can target each one of the three components indispensable for protein ubiquitination and subsequent degradation: the ubiquitinating machinery (E1, E2, and E3 enzymes), the protein substrate, and the proteasome (center of the circle). *S*-nitrosation can enhance the degradation rate of the protein substrate (point 1, e.g., *O* ^6^-alkylguanyl-DNA alkyltransferase, AGT) or its ubiquitination (point 2). Conversely, *S*-nitrosation can (i) inhibit ubiquitin ligase activity of several E3s, such as Parkin and XIAP (point 3), (ii) affect ubiquitination directly, by changing protein structure, as demonstrated for Bcl2 and FLIP (point 4), or indirectly, by inhibiting enzyme activities of proteins acting as positive modifiers of ubiquitination (e.g., IKK*β*, point 5), and (iii) directly impair protasome activity (point 6). *Red ring:* inhibitory effects; *green ring*: activating effects.

Nitric oxide can affect ubiquitin conjugation steps and proteasomal degradation of ubiquitinated proteins at different level (Figure 2). For instance, the catalytic site of ubiquitinating enzymes (E1-E2-E3) contains a cysteine residue that it is now arising could be susceptible to *S*-nitrosation [53–55]. *S-*nitrosation has been also shown to inhibit E3 ligase activity in RING (really interesting new gene) finger motif-containing proteins, modulating in such a way the downstream signaling cascades. Notably, RING E3s do not have recognizable active sites that define the “canonical” enzymes. Instead, they have large binding interfaces and act as scaffold proteins bringing together the participant E2 and substrate proteins. Therefore, *S*-nitrosation might affect the interacting properties of this class of E3 ligase. One example is the RING finger E3 ligase parkin, whose mutations have been demonstrated being implicated in Parkinson's disease etiopathogenesis. *S*-nitrosation of parkin inhibits its activity [54], thereby resulting in enhanced accumulation of protein aggregates, as well as impairment of autophagy-mediated removal of damaged mitochondria (mitophagy) [2] (Figure 2). Likewise, *S*-nitrosation of the RING finger E3 ligase X-linked IAP (XIAP) has been reported to inhibit ubiquitin-mediated proteasomal degradation of caspase 3, thereby resulting in the promotion of cell death by apoptosis [30, 55] (Figure 2).

Beside the effects on ubiquitin conjugating system, NO can directly interfere with the proteasome protein complex. In vascular smooth muscle cell, *S*-nitrosoglutathione (GSNO) exposure revealed that the 20S catalytic core of the 26S proteasome contains 10 cysteines which undergo *S*-nitrosation, thus resulting in the inhibition of all three catalytic activities of the complex (chymotrypsin-, trypsin-, and caspase-like) [56] (Figure 2). Since the 26S proteasome is responsible for the time-dependent degradation of cell cycle proteins (e.g., cdk2, cdk4, cyclins, and the cyclin-dependent kinase inhibitors p21 and p27) [57], the inhibition of its activity by *S*-nitrosation can affect cell cycle progression and proliferation. The effects of *S*-nitrosation-mediated modulation of protein turnover can also directly impact on target proteins, such as in the cases of the key apoptosis regulatory protein Bcl2 and the antiapoptotic FLICE inhibitory protein (FLIP), whose *S*-nitrosation inhibits their ubiquitination and proteasomal degradation and finally leads to apoptosis suppression [58, 59] (Figure 2). Furthermore, *S*-nitrosation may indirectly inhibit ubiquitination *via* the regulation of alternative posttranslational modifications, such as in the case of the NO-mediated activation of nuclear factor *κ*-light-chain enhancer of activated B cells (NF-*κ*B). *S*-nitrosation of I*κ*B kinase (IKK*β*) inhibits its kinase activity, making it unable to phosphorylate the inhibitor of NF-*κ*B (I*κ*B) which, in turn, does not undergo ubiquitination and degradation *via* the proteasome, leaving NF-*κ*B unable to translocate into the nucleus and to induce transcription [60, 61] (Figure 2).

### 2.3. Activating Effects of *S*-Nitrosation

While many observations argue for *S*-nitrosation being a posttranslational modification that negatively affects UPS efficiency, there is a literature supporting the hypothesis that it can also indirectly enhance ubiquitin-mediated protein degradation. In regards to this aspect, it should be reminded that polyubiquitination-mediated degradation of some proteins relies upon the conversion of N-terminal domain-located asparagine, glutamine or cysteine residues into arginine. This is required in order to allow recognition by E3 ligases of proteins being degraded (the so called *N-rule*) [62]. Arginylation of the N-terminal cysteines seems to be facilitated by *S*-nitrosation thereby suggesting the involvement of this modification in the turnover of many substrates.

The propensity of a number of proteins to undergo ubiquitination after *S*-nitrosation is not so unusual. An exhaustive example is provided by the key DNA repair protein *O* ^6^-alkylguanine-DNA alkyltransferase (AGT), whose *S*-nitrosation has been reported to induce its massive proteasomal degradation and to negatively affect DNA repair [63] (Figure 2).

## 3. Ubiquitin-Protein System and Protein *S*-Nitrosation in Neuromuscular Diseases

### 3.1. Muscular Atrophies and Myopathies

Skeletal muscle atrophy can be defined as wasting or decrease in muscle mass owing to injury, lack of use, or disease. Muscle atrophy arises either from damage to the nerves that supply the muscles (neuromuscular disease) or disease of the muscle itself (musculoskeletal disease). The causes of atrophy rely on genetic mutations, such as in amyotrophic lateral sclerosis and muscular dystrophies, or are derived from systemic diseases, such as diabetes, cancer, and metabolic inflammation [64]. Nevertheless, the totality of atrophic conditions shares an imbalance between protein synthesis and degradation, resulting in reduced protein synthesis and increased protein breakdown, which in turn leads to reduced muscle mass and muscle fiber size. Indeed, independently of the etiology, muscle atrophies are commonly identified by the upregulation of the same set of genes, the so-called atrophy-related genes, or *atrogenes*, among which atrogin-1/muscle atrophy F-box (MAFbx) and muscle RING finger 1 (MuRF1) are well documented. These genes belong to the family of E3 ligase enzymes, which are responsible for the massive protein breakdown occurring in these diseases. In skeletal muscle atrophy, the central role of the ubiquitin-proteasome pathway has been characterized through the pioneering studies on gene expression profile independently performed by the research groups of Goldberg and Glass [65, 66]. In particular, they revealed that atrogin-1/MAFbx and MuRF1 are the two muscle-specific ubiquitin ligases upregulated in different models of muscle atrophy and responsible for the increased protein degradation *via* the UPS.

Another interesting common point of muscular atrophies is the occurrence of nitroxidative stress [67, 68]; indeed, accumulation of nitrotyrosine adducts has been detected in models of disuse-induced atrophies, as well as genetic-based dystrophies [69]. In skeletal muscle, the maintenance of a fully functioning fiber requires the correct assembly of the dystrophin glycoprotein complex (DGC). It is composed by several transmembrane and peripheral accessory proteins which are highly expressed in the sarcolemma and constitute a critical link between the cytoskeleton and the extracellular matrix [70]. It has been reported that DGC participates in cell signaling through the involvement of nNOS, which is predominant muscular isoform of NOS found to be associated to the complex *via* the alpha-syntrophin [71]. One possible mechanism underlying the overproduction of NO in muscle cell under atrophic conditions is the dislocation of nNOS from the DGC underneath the sarcolemmal membrane, followed by its redistribution into the cytosol where it produces NO [72]. The majority of congenital dystrophies depends on mutations in any of the complex components [73]. Interestingly, the dislocation of nNOS occurs in many types of dystrophies, such as Duchenne muscular dystrophy [70], which is characterized by the complete ablation of dystrophin, and in autosomal recessive limb girdle muscular dystrophy (AR-LGMD), where mutations of sarcoglycan proteins seem to be the main causative events of the pathology [74]. Furthermore, dislocation of nNOS from the DGC occurs also in rat models of disuse- or denervation-induced atrophy, indicating that this mechanism could underlie, at least in part, the pathology of muscular disorders [72]. More recently, it has been also demonstrated that nNOS dislocation induces force reduction, which is typical feature of dystrophin-null mouse models, by means of still not elucidated mechanisms putatively involving tyrosine nitration and also *S*-nitrosation [75]. The first evidence of *S*-nitrosation involvement in this class of pathologies involves the *S*-nitrosation, and the subsequent hyperactivation, of the Ca^2+^ release channel ryanodine receptor 1 (RyR1). Such a modification leads to a chronic Ca^2+^ leakage from sarcoplasmatic reticulum [76, 77] and triggers mitochondrial fragmentation underlying muscle atrophy [78]. Moreover, NO has been reported being involved in the activation of Forkhead box O (FoxO) 3a transcription factor (FoxO3a). Although the molecular mechanisms underlying this process are not well established yet, the increase of intracellular NO levels within the cell seems to be capable of mediating FoxO3a activation and nuclear translocation, thereby inducing skeletal muscle atrophy by upregulating MuRF1 or atrogin-1/MAFbx [79, 80]. In this context, it is of note to remind that also myogenin, a protein involved in myofiber differentiation and development of functional muscles, has been reported undergoing *S*-nitrosation, reasonable at the level of Cys61 and Cys65 [81]. This modification profoundly impacts on myogenin ability to bind DNA at the promoter regions to activate downstream gene expression (e.g., caveolin-3) and finally result in muscle atrophy (Table 2).

### 3.2. Neuropathies

Many diseases affecting muscle health and function also induce peripheral neuropathies as side effects. Indeed, denervation or peripheral nerve injuries (e.g., those characterized by partial loss of fibers or myelin in the nerve) strongly contribute to muscle wasting [82]. Besides the already mentioned muscular dystrophies, also cancer and diabetes, as well as aging-related cachexia, show alterations of nerve physiology associated with NO dysbalance and PSNOs increase [72, 83]. Moreover, it has been reported that NO overproduction and *S*-nitrosation could be directly associated with the transduction pathways underlying fatigue and myalgia deriving from muscle wasting [84], which are typical features of skeletal muscle atrophic states. In particular, recent lines of evidence argue for *S*-nitrosation of the transient receptor potential vanilloid 1 and ankyrin 1 (TRPV1 and TRPA1, resp.), two polymodal ion channels of peripheral sensory dorsal root ganglia, being the principal event underlying the sensitivity of noxious stimuli impinging on peripheral nociceptors [85, 86] (Table 2).

As above reported for correct muscle maintenance, a balanced ratio between protein synthesis and degradation is important also for neuronal viability. Actually, an efficient removal of unfolded or damaged proteins and organelles is crucial to prevent neuronal death and to preserve axonal integrity. In regard to this aspect, several proteins have been reported playing a pivotal role in neurodegenerative diseases when *S*-nitrosated. Drp1 is a case in point, as its mitochondrial translocation and GTPase activity seem to be enhanced when the protein undergoes *S*-nitrosation at Cys644 [87]. This *gain-of-function* modification—which has been found associated with Alzheimer's disease and pathological conditions affecting central nervous system (Table 1)—alters mitochondrial dynamics process by increasing mitochondrial fragmentation and finally contributes to neuronal cell demise. We readily refer to other comprehensive and more focused reviews dissecting in detail this aspect [88], while attempting here to deal with how *S*-nitrosation of proteins involved in ubiquitination process can impact on peripheral nervous system physiology. Among them, the ubiquitin E3 ligase TRIM2 (tripartite motif containing protein 2), which is involved in the regulation of axonal specification and polarization [89], has been very recently proposed to be neuroprotective [90]. In particular, Ylikallio and colleagues reported that *TRIM2* mutations that result in the complete loss of the protein are associated with childhood onset of axonal neuropathy leading to muscle mass reduction. Mouse models of TRIM2 deficiency recapitulate the human phenotype due to an aberrant axonal accumulation of neurofilaments that are no more ubiquitinated and degraded *via* the proteasome [91]. Although no evidence on possible redox reactions, namely, *S*-nitrosation, have been provided yet on TRIM2, it is plausible that its occurrence could inhibit TRIM2 activity, as already demonstrated for many other members of the ubiquitin E3 ligase superfamily, thereby allowing speculating that the existence of an *S*-nitrosated form of TRIM2 could correlate with the onset of axonopathy and muscle atrophy-associated peripheral neuropathy. Likewise, the mitochondrial ubiquitin E3 ligase MITOL has been demonstrated to regulate mitochondrial dynamics, as well as to counteract the toxicity of polyglutamine-containing protein ataxin 3 [92] and mutant superoxide dismutase 1 [93], which are the main causes of Machade-Joseph disease and amyotrophic lateral sclerosis, respectively. Very recently it has been indicated that MITOL undergoes *S*-nitrosation and loss of activity [94], thereby resulting in mitochondrial aggregation and neuronal cell death. Among the large amount of substrates, MITOL also regulates the turnover of microtubule-associated protein 1B-light chain 1 (LC1) that, intriguingly, is ubiquitinated by MITOL and then subjected to proteasome-mediated degradation only when *S*-nitrosated [94]. Thus modified, indeed, LC1 translocates to the cytoskeleton, stabilizes microtubules and, consequently, freezes organelle transport. Therefore, under moderate (physiological) NO concentration, MITOL is required to maintain intracellular traffic by promoting LC1 degradation. Conversely, under nitrosative/toxic conditions, such as upon *N*-methyl-d-aspartate (NMDA) rector chronic activation, MITOL is inactivated, resulting in LC1 accumulation and mitochondrial dysfunction typical of neurodegenerative disease [94].

## 4. Future Research Perspectives and Therapeutic Strategies

On the basis of what previously described, excessive *S*-nitrosation seems to play a detrimental role in neurological disorders mostly due to its direct inhibitory effect on ubiquitin E3 ligases involved in the maintenance of cellular homeostasis (e.g., MITOL and TRIM2). Moreover nitrosative stress has been indicated to negatively affect muscle function and to induce muscular atrophy, owing to an excessive activation of the UPS (e.g., by means of atrogene induction *via* FoxO). In accordance with the above reported results, *S*-nitrosation has been also demonstrated being deeply implicated in sensitivity to nociceptive stimuli due to its impact on TRP ion channels. Altogether, these observations correlate with recent lines of evidence indicating that the sulfhydryl-containing molecule *N*-acetylcysteine (NAC) reduces pain and ameliorates muscle performance [95, 96], protects dystrophic myofibers against eccentric muscle damage, and contrasts abnormal calcium influx [97]. Being NAC a well-known antioxidant and denitrosating agent, this evidence suggests that nitrosative stress might represent a condition underlying or contributing to some pathological features of skeletal muscle disorders. Along this line, it has been demonstrated that pharmacological inhibition or genetic ablation of nNOS [75] reverts neuromuscular pathological phenotypes; however, these approaches have still not allowed discriminating whether tyrosine nitration or cysteine *S*-nitrosation is the principal mediator of neuropathy and myopathy induced by NO overproduction. Undoubtedly, the use of different NO donors does not represent a good model to unravel this issue. Indeed, their delivery of NO, which recapitulates a burst more than a persistent, and physiological, flux, has so far produced still questionable results. Cellular and mouse models of “genetically altered” *S*-nitrosation (e.g., GSNOR downregulating or knock-out models) could be of help in the next future to evaluate the specific contribution of different NO-mediated protein modifications: nitrationversus *S*-nitrosation. Figuring out this issue would open new avenues for the pharmacological treatment aimed at the restoration of a correct neuromuscular physiology for pathologies whose prognosis, on the contrary, is characterized by a progressive and irreversible loss of motion and cognitive abilities accompanied by chronic pain.

## Figures and Tables

**Table 1:** Examples of pathological conditions associated with alterations in Prot-SNOs.

Protein-SNO	Pathology	Reference
Dynamin-related protein 1	Alzheimer disease	Cho et al., 2009 [86]
Protein disulfide isomerase	Alzheimer disease	Uehara et al., 2006 [98]
	Parkinson disease	
X-linked inhibitor of apoptosis	Alzheimer disease	Nakamura et al., 2010 [30]
	Parkinson disease	
Parkin	Parkinson disease	Chung et al., 2004 [53]
Peroxiredoxin-2	Parkinson disease	Fang et al., 2007 [97]
Ryanodine receptor 2	Heart failure	Gonzalez et al., 2007 [99]
*O* ^ 6^-alkylguanine-DNA alkyl transferase	Cancer	Wei et al., 2010 [62]
Ryanodine receptor 1	Duchenne/limb-girdle muscular dystrophy	Bellinger et al., 2009 [76]
		Andersson et al., 2012 [100]

**Table 2:** Role and targets of NO in neuromuscular dysfunctions.

NO adduct	Protein	Reference
*S*-NO Cys-3635	Ryanodine receptor 1	Bellinger et al., 2009 [76]
Tyr-NO?	NFkB	Suzuki et al., 2007 [71]
Tyr-NO?	FoxO3	Suzuki et al., 2007 [71]
*S*-NO Cys-553/558	Transient receptor potential cation channel	Yoshida et al., 2006 [85]
*S*-NO Cys-61/65	Myogenin	Martínez-Moreno et al., 2008 [80]
